# Editorial: Nanoparticle-based delivery systems on immunomodulation

**DOI:** 10.3389/fimmu.2025.1626027

**Published:** 2025-06-17

**Authors:** Pedro Augusto Carvalho Costa, Pedro Pires Goulart Guimaraes

**Affiliations:** Department of Physiology and Biophysics, Institute of Biological Sciences, Federal University of Minas Gerais, Belo Horizonte, Minas Gerais, Brazil

**Keywords:** nanotechnology, nanoparticles, drug delivery, gene delivery, immunomodulation, immunotherapy, autoimmune diseases, cancer

Nanoparticles (NPs), typically ranging from 1 to 100 nanometers in size, have revolutionized biomedical research due to their unique physicochemical properties. Their structural diversity, stemming from compositions that include polymers, lipids, metals, oxides, and carbon enables tailored functionalities for applications ranging from diagnostics to drug delivery (1). Today, NPs occupy a central position in the development of advanced therapies, with one of their most transformative roles emerging in the field of immunology. NPs offer several key advantages over traditional platforms such as enhance antigen stability, facilitate lymph node targeting, promote efficient uptake by antigen-presenting cells, and mimic pathogen-associated structural features. These properties allow for finely tuned immune responses, with the capacity to modulate innate and adaptive immunity based on design parameters (2). The success of lipid nanoparticle (LNP)-based mRNA vaccines against COVID-19 validated decades of nanotechnology research and opened new avenues for vaccine design against infectious diseases, cancers, and autoimmune disorders. Looking ahead, the convergence of nanotechnology and immunology is opening a new frontier in biomedical applications, where engineered NPs have the potential to fundamentally transform disease prevention and treatment.

Hanna et al. described an intradermal injection of gold NPs (GNPs) loaded with the proinsulin peptide C19-A3 in type 1 diabetes patients leads to the recruitment and retention of immune cells in the skin, particularly clonally expanded T-cells with activated phenotypes (Hanna et al.). About half of these T-cells were found to be specific for either GNP or proinsulin. Gold-specific clones were all CD8+, while proinsulin-specific clones included both CD8+ and CD4+ cells, with CD8+ clones showing a cytotoxic phenotype marked by high levels of granulysin (GNLY) and KIR receptors. These antigen-specific T-cells persisted in the skin for months to years, exhibiting various memory phenotypes. The study suggests that the T-cell response, which targets both the gold core and the antigen, could enhance the formation of resident memory T-cells in response to vaccines. Furthermore, their scRNAseq data indicates that focusing on these expanded T-cells is an effective method for identifying antigen-specific cells, which could aid in monitoring the intradermal delivery of antigens and NPs for immune modulation in humans.

Su et al. developed a vaccine against a major pathogen causing severe diarrhea and high mortality in newborn piglets using bacterium-like particles (BLPs) called S1-BLPs, which display the S1 protein from the Porcine epidemic diarrhea virus (PEDV) spike protein (Yin et al.). The study examined how different vaccination routes affected immune responses in mice. Results showed that intramuscularly vaccinated mice had higher serum IgG levels compared to those vaccinated intranasally. However, intranasal vaccination led to the presence of specific IgA antibodies in both serum and intestinal fluid, which were absent in intramuscularly vaccinated mice. Additionally, intranasal administration increased cytokines IFN-γ and IL-4 levels in serum. The immune response from intramuscular injections indicated a stronger type 1 helper T (Th1) cell immunity. Overall, S1-BLPs can induce both systemic and local immune responses, with the route of administration influencing antibody types and Th responses. Intranasal S1-BLPs effectively stimulated secretory IgA in the intestinal mucosa, suggesting their potential as a mucosal vaccine against PEDV.

Yin et al. developed an innovative virus-like nanoparticle (VLP) vaccine that simultaneously displayed Nipah virus (NiV) attachment glycoproteins (G) from NiV and related henipaviruses, leveraging the self-assembling characteristics of ferritin protein (Adugna et al.). When compared to the NiV G subunit vaccine, their NP vaccine generated significantly higher levels of neutralizing antibodies and offered complete protection against a lethal NiV infection in Syrian hamsters. Notably, the NPs vaccine prompted the production of antibodies that showed enhanced cross-reactivity to both related and unrelated henipaviruses. These results highlight the promising potential of ferritin-based NP vaccines in delivering broad-spectrum and long-lasting protection against Nipah virus and emerging zoonotic henipavirus threats.

In a review paper, Adugna et al. described that vaccination remains the primary strategy to combat Japanese encephalitis (JE), with inactivated and live attenuated vaccines demonstrating strong immunogenicity (3). However, traditional vaccine production is costly and involves significant biosafety risks due to the extensive cultivation of pathogens, posing additional dangers for individuals with compromised immune systems. As a result, research has shifted toward NP platforms for developing next-generation JE vaccines. A systematic review analyzed 28 studies on NP-based JE vaccines published between 2000 and 2023, revealing that 57.14% focused on virus-like particles (VLPs) using structural proteins, while other studies employed sub-viral particles (SVPs), biopolymers, and synthetic or inorganic NPs. These vaccines consistently enhanced humoral and cellular immune responses, providing 70–100% protection against lethal JE virus challenges in mice. With further refinement, NP-based vaccines may offer promising solutions for immunizing humans and animals.

**Figure 1:**
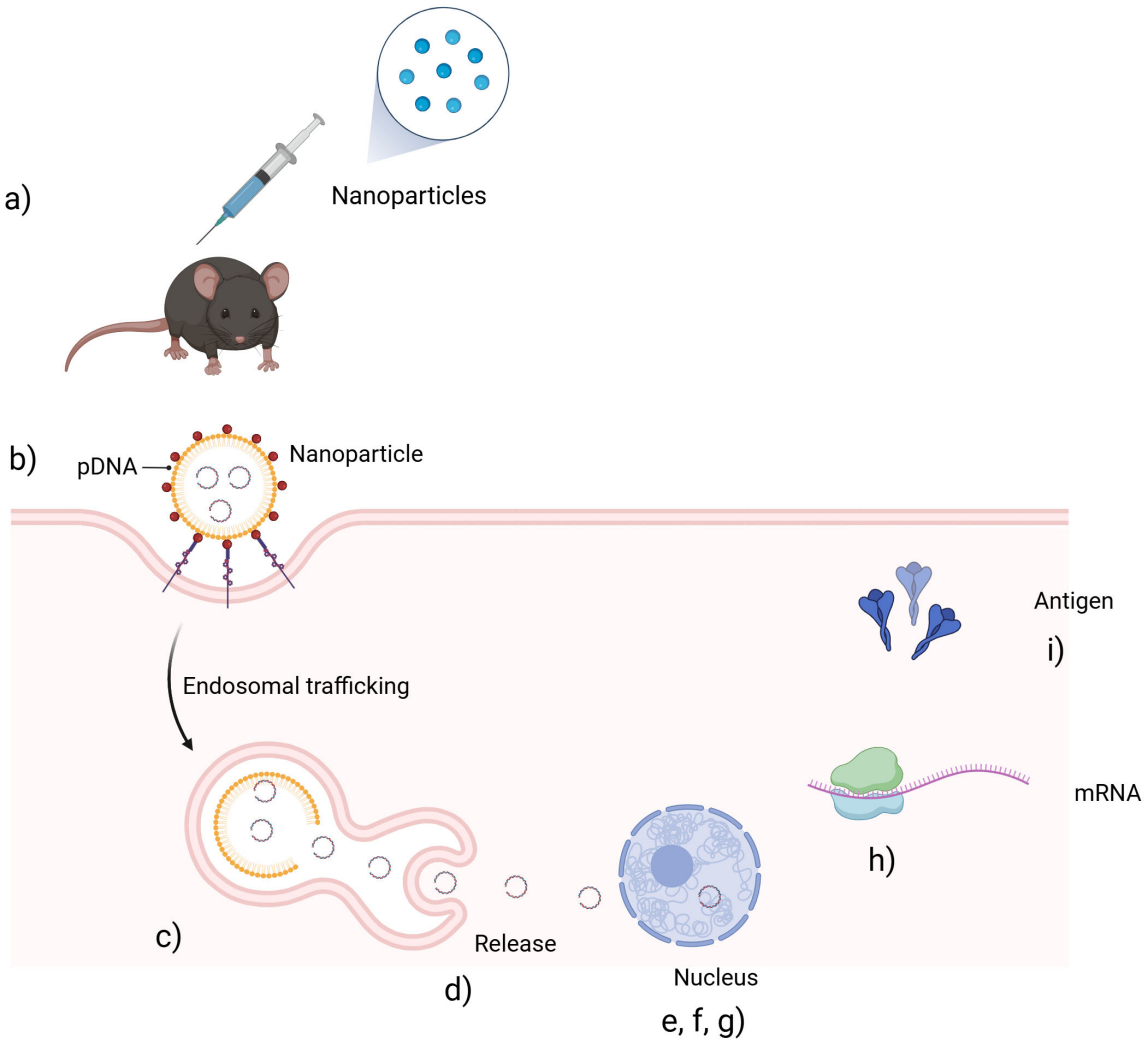
Schematic representation of the preparation and *in vivo* delivery of a nanoparticle-based plasmid DNA (pDNA) vaccine. **(a)** The NP-based pDNA vaccine is administered via injection. **(b)** Nanoparticles are taken up by target cells through endocytosis. **(c)** The vaccine enters endosomes. **(d)** Nanoparticles escape the endosomal compartment. **(e, f, g)** pDNA is released into the cytoplasm, enters the nucleus and transcription of pDNA into mRNA occurs. **(h)** mRNA is translated into antigenic proteins in the cytoplasm. **(i)** Antigens are produced and presented to initiate an immune response.

Recent technological advances—particularly in nanoparticle (NP)-based delivery systems—have significantly accelerated the production of safe, targeted, and adaptable immunotherapies (4). NP-based technologies offer a compelling solution, enabling precision targeting, controlled release, and immune system modulation. Platforms such as mRNA, protein-based, and DNA technologies have successfully developed promising antiviral solutions (**Figure 1**) (5–7). Beyond infectious diseases, NP platforms are expanding the possibilities of immunotherapies across a wide range of conditions. Recent studies have demonstrated the versatility of NPs in enhancing immune responses: Hanna et al. used gold nanoparticles (GNPs) to deliver proinsulin peptides, boosting antigen-specific T-cell responses in type 1 diabetes; Su et al. employed bacterium-like particles (BLPs) displaying PEDV S1 proteins to successfully induce mucosal and systemic immunity through intranasal vaccination; and Yin et al. developed ferritin-based virus-like particles (VLPs) for Nipah virus, achieving robust neutralizing antibody responses and cross-reactivity (Adugna et al.). These findings collectively illustrate the transformative potential of NP platforms in immunotherapies and vaccine design, with broad implications for combating infectious diseases, autoimmune disorders, and beyond.
